# Continuous performance test impairment in a 22q11.2 microdeletion mouse model: improvement by amphetamine

**DOI:** 10.1038/s41398-018-0295-3

**Published:** 2018-11-14

**Authors:** Simon R. O. Nilsson, Christopher J. Heath, Samir Takillah, Steve Didienne, Kim Fejgin, Vibeke Nielsen, Jacob Nielsen, Lisa M. Saksida, Jean Mariani, Philippe Faure, Michael Didriksen, Trevor W. Robbins, Timothy J. Bussey, Adam C. Mar

**Affiliations:** 1 Department of Psychology, University of Cambridge, Cambridge, UK; 2 MRC and Wellcome Trust Behavioural and Clinical Neuroscience Institute, University of Cambridge, Cambridge, UK; 3 Neuroscience Institute, New York University Medical Center, New York, NY USA; 4 Department of Neuroscience and Physiology, School of Medicine, New York University, New York, NY USA; 5 School of Life, Health and Chemical Sciences, The Open University, Walton Hall, Milton Keynes, UK; 6 Fatigue and Vigilance team, Neuroscience and Operational Constraints Department, French Armed Forces Biomedical Research Institute (IRBA), Brétigny-sur-Orge, France; 7 VIFASOM team (EA 7330), Paris Descartes University, Sorbonne Paris Cité, Hôtel Dieu, Paris, France; 8 Sorbonne Universités, Université Pierre et Marie Curie (UPMC), CNRS, INSERM, U1130, Institut de Biologie Paris Seine (IBPS), UMR 8246 Neuroscience Paris Seine (NPS), Team Neurophysiology and Behavior, Paris, France; 9 Sorbonne Universités, Université Pierre et Marie Curie (UPMC), CNRS, Institut de Biologie Paris Seine (IBPS), UMR 8256 Biological adaptation and ageing (B2A), Team Brain Development, Repair and Ageing, Paris, France; 10 APHP Hôpital, DHU Fast, Institut de la Longévité, Ivry-Sur-Seine, France; 11 H. Lundbeck A/S, Synaptic Transmission, Neuroscience Research DK, Copenhagen, Denmark; 12 Molecular Medicine Research Group, Robarts Research Institute & Department of Physiology, Western University, London, ON Canada; 13 Pharmacology, Schulich School of Medicine & Dentistry, Western University, London, ON Canada; 14 The Brain and Mind Institute, Western University, London, ON Canada

## Abstract

The 22q11.2 deletion syndrome (22q11.2DS) confers high risk of neurodevelopmental disorders such as schizophrenia and attention-deficit hyperactivity disorder. These disorders are associated with attentional impairment, the remediation of which is important for successful therapeutic intervention. We assessed a 22q11.2DS mouse model (Df(h22q11)/+) on a touchscreen rodent continuous performance test (rCPT) of attention and executive function that is analogous to human CPT procedures. Relative to wild-type littermates, Df(h22q11)/+ male mice showed impaired attentional performance as shown by decreased correct response ratio (hit rate) and a reduced ability to discriminate target stimuli from non-target stimuli (discrimination sensitivity, or d’). The Df(h22q11)/+ model exhibited decreased prefrontal cortical-hippocampal oscillatory synchrony within multiple frequency ranges during quiet wakefulness, which may represent a biomarker of cognitive dysfunction. The stimulant amphetamine (0–1.0 mg/kg, i.p.) dose-dependently improved d’ in Df(h22q11)/+ mice whereas the highest dose of modafinil (40 mg/kg, i.p.) exacerbated their d’ impairment. This is the first report to directly implicate attentional impairment in a 22q11.2DS mouse model, mirroring a key endophenotype of the human disorder. The capacity of the rCPT to detect performance impairments in the 22q11.2DS mouse model, and improvement following psychostimulant-treatment, highlights the utility and translational potential of the Df(h22q11)/+ model and this automated behavioral procedure.

## Introduction

A copy number variant (CNV) composed of a hemizygous microdeletion at chromosomal locus 22q11.2 confers large genetic risk for schizophrenia^1^, attention-deficit hyperactivity disorder (ADHD)^2^ and autism^3^. The 22q11.2 microdeletion syndrome (22q11.2DS) and its related neuropsychiatric disorders are associated with executive and attentional impairments^4^. These deficits are of central interest for translational^5^ and genetic studies^6^ aimed at discovering more effective therapeutics.

Attentional and executive dysfunctions are commonly evaluated using computerized continuous performance tests (CPTs)^7^. Typically, visual target or non-target stimuli are briefly presented at a fixed screen location across a series of continuous, sequential trials. The subject is required to rapidly respond to targets and withhold from responding to non-targets. Non-affected individuals with high genetic load of schizophrenia-related genetic variants^8^, and 22q11.2 deletion carriers^7,9–12^, show impaired CPT performance. These impairments predict functional outcome^11,13^, appear independent of general intelligence^9^, and are often unaffected by available therapeutics^14^. Deficits in CPT performance are therefore important targets for therapeutic discovery efforts.

Several mouse models of 22q11.2DS have been generated^15–19^. Studies investigating the performance of these models across multiple cognitive domains, including associative and spatial learning, flexibility, and memory, have yielded equivocal results^19–23^. One of the consistent behavioral impairments in these models is an acquisition deficit on a T-maze delayed non-match to position task^19,23–25^. This deficit has been commonly ascribed to an impairment in working memory and has been linked to prefrontal cortical (PFC)-hippocampal asynchrony within theta and gamma bands^20,24^. However, the T-maze impairment in the 22q11.2DS mouse model appears transient and delay-independent—inconsistent with a primary deficit in working memory^23^. PFC-hippocampal synchrony is also associated with executive function and/or attentional processes in other behavioral paradigms^26–28^.

There is a paucity of studies evaluating attentional function in 22q11.2DS mouse models^29^. One recent study assessed *divided visuospatial attention* using the 5-choice serial reaction time task (5-CSRTT) and reported either no effect, or paradoxically improved performance after extended training, in the Df(h22q11)/+ model^23^. However, to date, there have been no assessments of *focused visual attention* in 22q11.2DS mouse models. In 22q11.2 deletion carriers, assessments by CPTs and related paradigms demonstrate clinically-relevant impairments on measures of correct response ratio (hit rate) and ability to discriminate target stimuli from non-target stimuli (signal detection sensitivity, or d’)^7,9–12^.

As part of the NEWMEDS initiative (Innovative Medicines Initiative Grant Agreement No. 115008), the current study assessed executive and attentional function in two cohorts of a 22q11.2DS mouse model (Df(h22q11)/+) and wild-type littermates. We evaluated aspects of focused attention and inhibitory control using a touchscreen rodent continuous performance test (rCPT) that has been developed to closely emulate the human paradigm^30,31^. The rCPT is experimenter-paced and features multiple complex luminance-matched target and non-target stimuli that require detection and discrimination as well as response inhibition^31^. We hypothesized that the rCPT would be sensitive for identifying attentional impairment in the Df(h22q11)/+ model as measured by d’ and/or hit rate. To further characterize the Df(h22q11)/+ mouse model, we investigated PFC-hippocampal coherence which has been proposed as an endophenotype of several neuropsychiatric disorders associated with 22q11.2DS, including schizophrenia^32^. PFC-hippocampal synchrony has been shown to be disrupted in another 22q11.2DS mouse model (Df(16)A+ /−) while animals are performing a maze task^20,24^. We probed the robustness and generalizability of this potential endophenotype by evaluating PFC-hippocampal synchrony in a separate cohort of Df(h22q11)/+ mice under quiet-wake “baseline” conditions, independent of potentially confounding influences of prior cognitive training or ongoing behavioral performance. Finally, we assessed the effect of acute systemic modafinil and amphetamine treatments in the (Df(16)A+ /−) model on rCPT performance. The behavioral effects of these drugs have been shown to diverge depending on dose to produce characteristic U-shaped response curves^33^ with beneficial effects of acute low-dose amphetamine or modafinil frequently being reported on tests of attention and response control in both humans and experimental animals^34–36^. Moreover, the psychostimulant methylphenidate has been demonstrated to acutely improve discrimination sensitivity, d’^37^, decrease target omissions and increase hits^38^ on CPTs in individuals with 22q11.2DS. Based on this evidence we hypothesized that both amphetamine and modafinil would improve d’ and/or hit rate of the Df(h22q11)/+ model in the rCPT.

## Method

### Animals

**Fig. 1:**
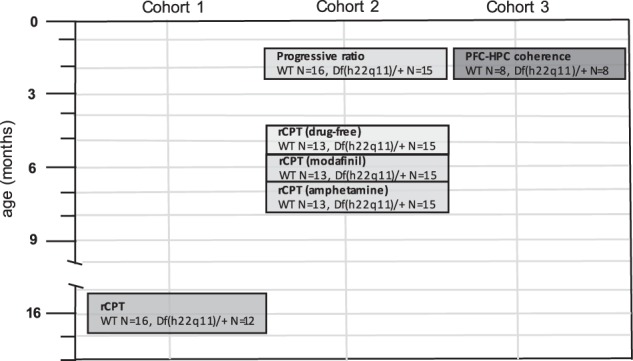
Timeline illustrating the experimental treatments and ages of the three cohorts of animals assessed in these experiments. See Methods for further description of these cohorts

The generation of Df(h22q11)/+ mice is described elsewhere^39^. Animals for these experiments were generated by mating wild-type C57BL/6N females with hemizygotic Df(h22q11)/+ males. Young (7–8 weeks), male Df(h22q11)/+ and wild-type littermate offspring were randomly selected and shipped to Cambridge and UPMC for experimentation. Figure 1 depicts the experimental timeline of this study. The behavioral experiments were performed at the University of Cambridge and used two cohorts of male mice housed as previously described^23^. Sample sizes were selected based on previous rCPT experiments^30^ and similar touchscreen paradigms^40^. One cohort of young-adult mice was trained on a progressive ratio (PR) paradigm (aged 9 weeks at start of testing; wild-type *N* = 16, Df(h22q11)/+ *N* = 15) and subsequently tested on the rCPT (aged 21 weeks at start of testing, wild-type *N* = 13, Df(h22q11)/+ *N* = 15). Another cohort of older mice (aged 16 months at the start of rCPT testing; wild-type *N* = 16, Df(h22q11)/+ *N* = 12) was assessed on the rCPT after extensive prior cognitive testing^23^. Animals were food restricted to about 85% of their free-feeding weight prior to behavioral testing. The electrophysiological studies were performed at UPMC Paris and used 16 male mice aged 3–7 months at testing (wild-type N = 8, Df(h22q11)/+ *N* = 8). All experiments were conducted in accordance with the European Union regulation (directive 2010/63 of 22 September 2010) and the UK Animals (Scientific Procedures) Act 1986.

### Drugs

Modafinil (Eli Lilly, USA; 0, 0.4, 4.0, 40 mg/kg, i.p, 30 min pretreatment time) was dissolved in vehicle (0.9% sterile saline and 0.5% arabic gum). d-Amphetamine sulphate (Sigma Aldrich, UK; 0, 0.25, 0.5, and 1.0 mg/kg, i.p; 20 min pretreatment time) was dissolved in vehicle (0.9% sterile saline). Dosing protocols were based on previous unpublished and published experiments^41–43^.

### Procedure

#### Behavioral procedures

See Supplementary Material for video clips of the apparatus and of animals performing the touchscreen rCPT and PR.

#### Apparatus

The apparatus is described elsewhere^40^. Briefly, the experiments used touchscreen chambers (Campden Instruments, UK) controlled via commercial (PR; ABET II, Lafaytte Instruments, USA) or in-house software (rCPT; VB.NET 2010, by A.C.M.). The PR task used a 5-aperture mask and the rCPT used a 3-aperture mask as described elsewhere^30,41^. Animals were trained to approach the touchscreen as detailed previously^40^.

#### The rodent continuous performance test

##### Training—stage 1 (white-square)

The rCPT training procedure is described in detail elsewhere^30,31^. Briefly, each trial began with a 2 s inter-stimulus interval (ISI) prior to stimulus presentation. To discourage superfluous responding to the screen, the ISI restarted if the subject touched the stimulus window during the ISI (‘ISI touch’). After the ISI, a white-square stimulus was presented for a 10 s stimulus duration (SD). If the animal touched the stimulus window within the ‘limited hold’ (LH) period after stimulus onset (LH:10.5 s), a reward (20 μl strawberry milkshake) was delivered coupled with white-noise (1 s) and magazine light illumination. Following the LH period (non-rewarded trials) or reward collection (rewarded trials), the next ISI was initiated. Trials were presented continuously until the session/phase criterion of 60 rewards was reached (one session for all animals).

##### Training—stage 2 (1-stimuli)

The correct stimulus (CS+: vertical or horizontal lines, counterbalanced across genotypes) was presented for a 5 s SD (LH:5.5 s). A 5 s delay to allow for reward consumption was added following reward collection. Other parameters remained identical to stage 1. All animals achieved criterion in a single session.

##### Training—stage 3 (2-stimuli)

On each trial, the mouse was presented with either the CS+ or a novel incorrect stimulus (CS−). The CS+ was identical to stage 2 while the CS− was a ‘snowflake’ stimulus^30^. The SD was reduced to 2.5 s (LH:2.5 s), the ISI was increased to 5 s and the CS+ probability was 50%. After a response to the CS−, a correction trial was implemented where the CS− was presented again following the ISI. Correction trials were presented until the animal successfully omitted a response to the CS−. The session ended after 100 correct responses or 45 min, whichever occurred first. Other parameters remained the same as in stage 2. The animals progressed as a group to the baseline rCPT procedure after 5 sessions on stage 3. All animals were performing at d’ greater than 0.6 criterion^30^.

##### Baseline rCPT (5-stimuli)

On each trial, animals were presented with one of five stimuli: four non-targets and the stage 3 target^30^. Other parameters remained identical to stage 3. Animals in the younger cohort were assessed for 6 sessions prior to acute, systemic treatment with modafinil and then amphetamine, using randomized Latin-square designs. The older cohort was assessed on baseline rCPT for 2 sessions followed by a series of probe tests (See Supplementary Material).

#### Progressive ratio

As motivational capacity can influence cognitive task performance, we also assessed Df(h22q11)/+ mice in a progressive ratio (PR) task. Animals in the younger cohort were tested in a touchscreen PR task designed to assess motivation through response requirements that increase according to linear ramp schedules (PR4-PR16) which is described elsewhere^41^.

#### Electrophysiology

##### Surgery

Mice were anesthetized with ketamine/xylazine and placed in a stereotaxic frame. Anesthesia was maintained with 3% isoflurane. Bipolar stainless steel electrodes were implanted bilaterally at coordinates relative to bregma in the infralimbic/prelimbic area of the PFC (dorsal-ventral: −1.55, anterior-posterior: +1.6, medial-lateral: ±0.5 mm) and CA1 region of the dorsal hippocampus (dorsal-ventral: −1.20, anterior-posterior: −1.94, medial-lateral: ± 1.2 mm). Monopolar ground electrodes were laid over the cortical layer of the cerebellum (anterior-posterior: + 6.24, medial-lateral: ± 1.0 mm) and olfactory bulb (anterior-posterior: + 4.2, medial-lateral: ±0.5 mm). Electrodes were fixed to the skull with dental acrylic and connected to an electrode interface board (8 channel headstage EIB-8; Neuralynx, USA). Antiseptic (Povidone-iodine) and local anesthetic (lidocaine) solutions were applied post-surgery. Animals were permitted to recover until regaining pre-surgery body weight.

##### Signal recording

Recordings were done as previously described^44^. Briefly, recordings were done in animals using chambers that limited, but did not restrain movement, and were electrically and acoustically insulated and isolated from odors and the experimenters. A cold light (100lux) was placed 20 cm in front of the animal. This environment was used to minimize the known modulatory effects of spontaneous motor activity on hippocampal local field potentials (LFP)^45^. Animals were gradually acclimated to the recording set-up and procedure. Wild-type and Df(h22q11)/+ mice were assessed simultaneously (4 mice per genotype) using a Latin-square design and recordings were made at the same time each day to minimize circadian LFP effects. Baseline LFP recordings were obtained over 60 min using a Digital Lynx SX (Neuralynx) and were acquired with a cheetah32 data acquisition system (Neuralynx). No attempts to escape or notable stress reactions were observed (i.e. defecation, urination, freezing) during the recording sessions.

##### Signal analysis

Data were analyzed using Matlab (MathWorks®, USA) built-in functions and the Chronox toolbox^46^. LFPs were (i) acquired at 1000 Hz and offline band-pass filtered at 0.1–100 Hz with zero-phase shift filter function (zero-phase digital filtering *filtfilt* function), and (ii) de-rendered using local linear regression (*locdetrend* function from the Chronux toolbox:^46^ window-size 1 s, overlap 0.5 s) to remove slow drifts, and (iii) notch-filtered (*iirnotch* function) with notch located at 50 Hz to remove possible power line noise. The LFP signal was expressed in z-score units. The z-score normalization used the mean and the standard deviation from baseline (entire rest session) of each electrode. Power spectral density (PSD) of LFP data was calculated using the multitaper *spectrogram* method from the Chronux toolbox with time-bandwidth product of 5 and 10 slepian sequences of orthogonal data tapers (window-size 5 s, 2 s overlap). PSD was averaged over two similar brain regions (right and left hemisphere) for each frequency and time-bin. The multitaper *coherogram* method was used to calculate coherence (normalized spectral covariance) between the LFP from two structures with time-bandwidth product of 30 and 60 slepian sequences of orthogonal data tapers using a 30 s window-size without overlap. The signal was bandpass-filtered to extract theta oscillations by applying a 5–10 Hz finite impulse response bandpass with zero-phase shift filter function (*filtfilt* function).

### Statistical analysis

#### Behavior

rCPT hit rate was calculated as the ratio of target responses to target presentations. False alarm rate was calculated as the ratio of non-target responses to non-target presentations. The performance was evaluated using the signal detection measures of discrimination sensitivity (d’) and response criterion (c)^47^. The discrimination sensitivity index (d’) assesses the subject’s capacity to distinguish the target from the non-target stimuli. The response criterion index (c) assesses the subject’s propensity or willingness to respond to any stimulus (e.g., target or non-target). Discrimination sensitivity d’ was calculated as^48^

1 $$d\prime = z\left( {hit\,rate} \right) - z\left( {false\,alarm\,rate} \right)$$

with higher values denoting better ability to discriminate between target and non-target stimuli. Response criterion c was calculated as^48^

2 $$c = - 0.5\left( {z\left( {hit\,rate} \right) + z\left( {false\,alarm\,rate} \right)} \right)$$

with higher values denoting decreased responding to both target and non-target stimuli. ISI touch rate was calculated as the number of touches to the response window during the ISI divided by the total ISI time in minutes. Incorrect and correct response latency and reward latency were also collected. Sessions were further split into 50-trial bins and dependent variables were calculated within each bin. The measures in the PR test were break-point (defined as the number of stimulus responses made in the last successfully completed trial in a session), total touches, total trials, time-out time, and ‘blank’ touches (defined as responses to the four never-illuminated response locations) per minute^41^. The experimenter was not blinded to the genotypes/drug-treatments. However, all behavioral data acquisition and analysis were fully automated with no experimenter involvement. Drug-free rCPT and PR data were analyzed by mixed-model ANOVAs with genotype as the between-subjects factor and session, SD, ISI, target probability or stimulus contrast as within-subjects factors. To analyze our *a priori* hypothesis that the deficits in rCPT performance observed in Df(h22q11)/+ mice could be ameliorated by modafinil or amphetamine, the pharmacological data were analyzed specifically in Df(h22q11)/+ mice using one-way ANOVAs with dose as independent within-subjects factor. To assess the overall effect of these compounds across all animals, the data were also analyzed across both genotypes using mixed-model ANOVAs with genotype as a between-subjects factor and dose as a within-subjects factor. Dose-response patterns were also tested for linear and U-shaped (quadratic) effects^31^. Significant interactions and dose-response patterns were followed by simple main effect comparisons using one-way ANOVA.

#### Electrophysiology

Three bands of the PSD were analyzed for each structure: 0.1–3 Hz (delta), 6–12 Hz (theta) and 30–80 Hz (gamma). All datasets were tested for normality using Shapiro-Wilk tests. For multiple comparisons of normally distributed data we used mixed-model ANOVAs (e.g., with frequency bands and genotype as factors). For data with non-Gaussian distributions, we used non-parametric Friedman tests. Post-hoc tests (independent-samples *t*-tests or Wilcoxon rank-sum test) were performed to compare genotype PSD and coherence estimates to identify frequency bands differing in spectral analysis. The stepwise Holm-Bonferroni (H-B) algorithm was used to correct for family-wise error rate (i.e., potential interference during multiple comparisons) by ordering *p*-values and adjusting significance level α. Standard error (SEM) intervals were calculated through a jackknife method^46^.

## Results

### The rodent continuous performance test

**Fig. 2:**
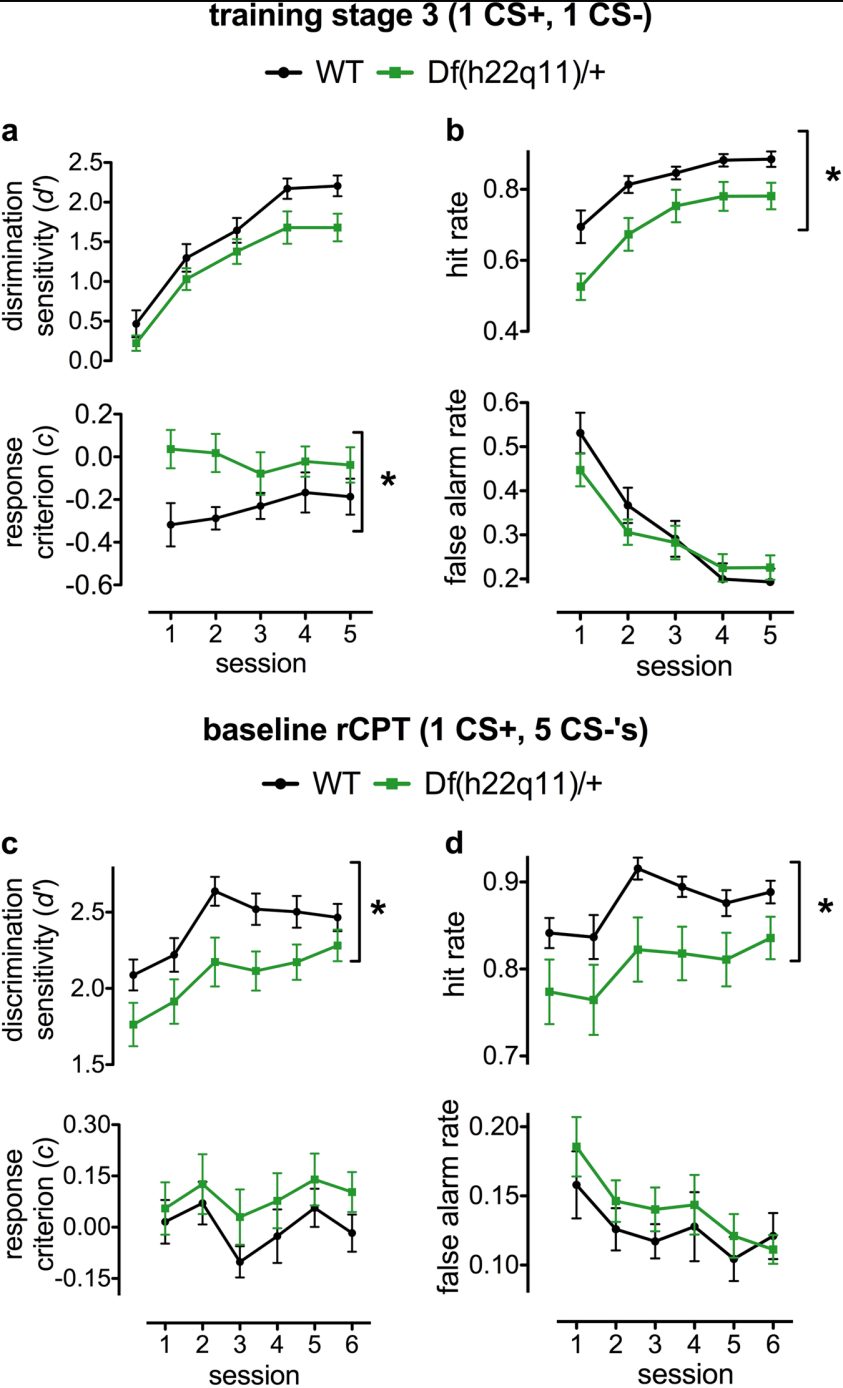
Performance of Df(h22q11)/ + and wild-type littermates on the 2-stimulus training stage 3 and the baseline 5-stimulus rCPT. Performance of Df(h22q11)/ + and wild-type littermates on the 2-stimulus training stage 3 and the baseline 5-stimulus rCPT). Data are presented as means ± SEM. Discrimination sensitivity (*d’*) is an index of the subject’s ability to distinguish target from non-target stimuli, while response criterion (*c*) describes the subject’s propensity to respond to any stimulus**. a** 2-stimulus: d’ and c. Df(h22q11)/+ mice had increased response criterion c. Df(h22q11)/+ mice showed a non-significant decrease in d’ (*p* = 0.051) relative to littermate controls. **b** 2-stimulus: hit rate and false alarm rate. Df(h22q11)/+ mice had decreased hit rates relative to controls. There was no effect of genotype on false alarm rate. **c** 5-stimulus rCPT: d’ and c. Df(h22q11)/+ mice had decreased d’ relative to controls. There was no effect of genotype on response criterion. **d** 5-stimulus rCPT: hit rate and false alarm rate. Df(h22q11)/+ mice showed decreased hit rate relative to controls. There was no effect of genotype on false alarm rate. Asterisk denotes significant effect of genotype (*p < 0.05)

See Supplementary Tables S1–S5 for detailed statistical analysis. rCPT performance of young (aged 21 weeks at the start of testing) Df(h22q11)/+ and wild-type mice is presented in Fig. 2a–d. Training stages 1 and 2, during which no non-target stimuli were presented, did not reveal any effects of genotype (Table S1). When a single, non-target stimulus was introduced, the Df(h22q11)/+ mouse exhibited a near-significant decrease in discrimination sensitivity (d’) (Fig. 2a; F_1,26_ = 4.203, *p* = 0.051), a significantly decreased hit rate (Fig. 2b; F_1,26_ = 9.552, *p* = 0.005) and an increased response criterion (c) (Fig. 2a; F_1,26_ = 6.971, *p* = 0.014) relative to wild-type littermate controls. On the baseline 5-stimulus rCPT, the Df(h22q11)/+ model showed decreased d’ (Fig. 2c; F_1,26_ = 5.724, *p* = 0.030) and decreased hit rate (Fig. 2d; F_1,26_ = 4.578, *p* = 0.042) compared to wild-type littermate controls. Time-bin analysis showed that Df(h22q11)/+ mice exhibited impairments throughout the session (data not shown).

A second older cohort of Df(h22q11)/+mice (aged 70 weeks at start of testing), with extensive previous cognitive testing experience, also showed decreased target hit rates when challenged with shorter stimulus durations (Supplementary Fig. S1a–b; genotype × SD: F_5,130_ = 4.795, *p* < 0.0001) and increased response criterion c when challenged with longer ISI times (Supplementary Fig. S2c–d; genotype × ISI: F_2,50_ = 3.221, *p* = 0.048). See Supplementary Material and Results from this cohort of Df(h22q11)/+ mice when tested on a range of different probe tests.

### Electrophysiological recordings

**Fig. 3:**
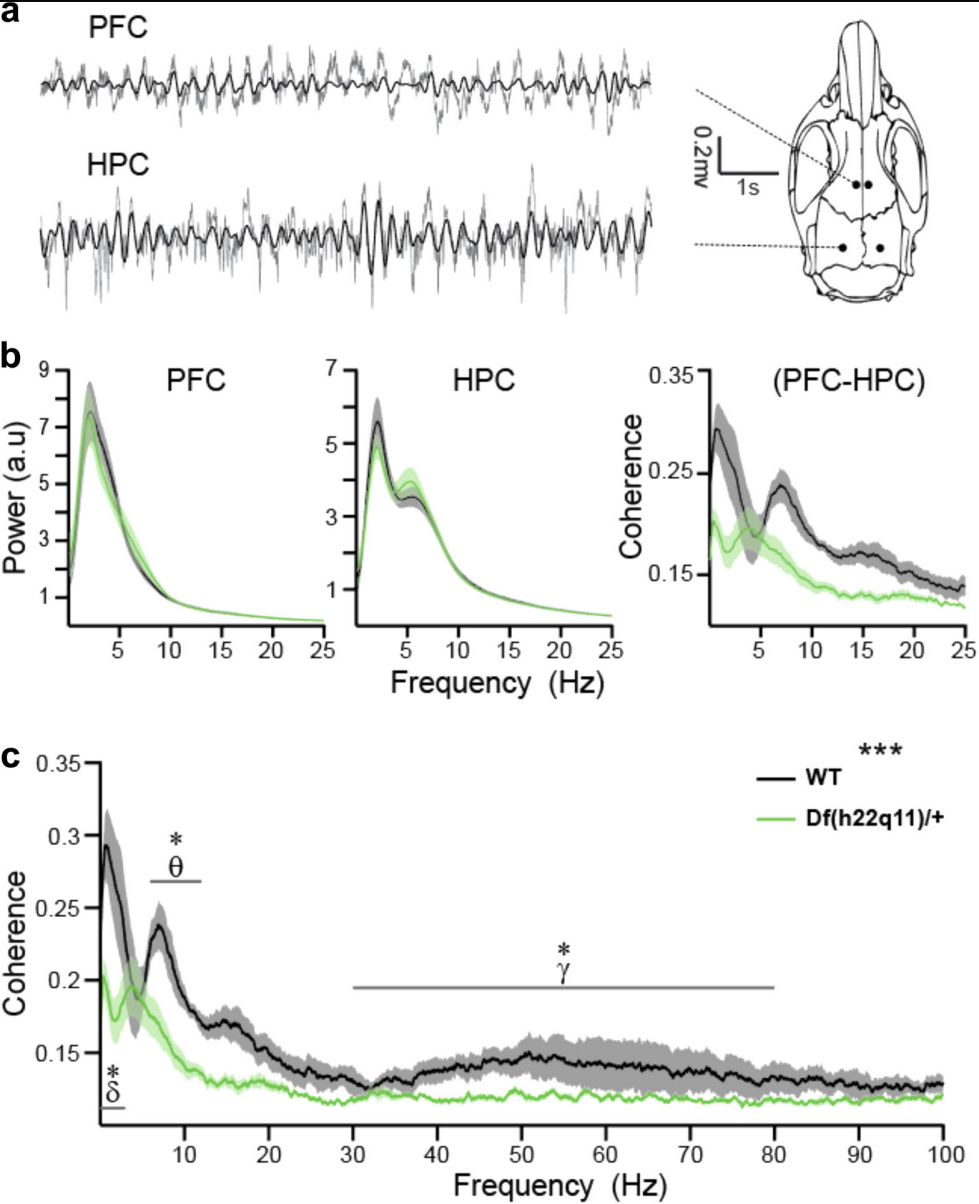
PFC and hippocampal synchrony and power spectra of Df(h22.q11)/+and wild-type littermates. Data are presented as mean ± SEM. Shaded areas represent SEM. **a** Left: Representative traces of the LFP recorded from the same animal simultaneously in the PFC and dorsal hippocampus during steady state conditions. Raw traces are plotted in gray and theta-filtered traces are overlaid in black. Right: Schematic diagram showing locations of LFP recording. **b** Averaged power spectra for each structure (PFC: left, HPC: middle) and average PFC-hippocampal coherence in 0.1–25 Hz range (right) in Df(h22.q11)/+ and wild-type littermates. **c** Average PFC-hippocampal coherence in 0.1–100 Hz range (same as b). Asterisk denotes significant effect of genotype (*p < 0.05, **p < 0.005, ***p < 0.0005)

PFC-hippocampal coherence data are presented in Fig. 3. See Supplementary Figures S2–S4 for additional analyses. Representative PFC and hippocampal LFP traces are shown in Fig. 3a. PFC-hippocampal coherence was reduced in Df(h22q11)/+ mice (Fig. 3c; genotype: F_1,36_ = 16.190, *p* < 0.001). Post-hoc analyses showed PFC-hippocampal coherence reductions in the delta (*p* = 0.030, *t*-test, H-B corrected) theta (*p* = 0.0027, t-test, H-B corrected) and gamma (*p* = 0.0035, Wilcoxon rank-sum, H-B corrected) bands of Df(h22q11)/+ mice. Genotype did not affect LFP frequency contents in the PFC (Fig. 3b, left; χ^2^ = 0.09, *p* = 0.762, Friedman ANOVA) or hippocampus (Fig. 3b, middle; F_1,42_ = 7.04, *p* = 0.601, two-way ANOVA).

### Effects of pharmacological interventions on continuous performance

#### Modafinil in the Df(h22q11)/+ model

**Fig. 4:**
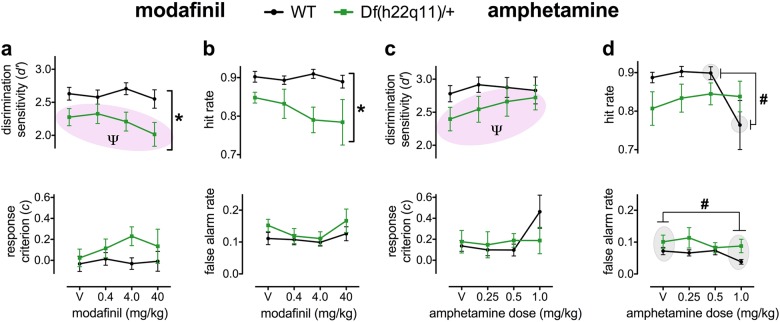
Performance of Df(h22q11)/ + and wild-type littermates on the 5-stimulus rCPT when treated with acute systemic modafinil and amphetamine. Performance of Df(h22q11)/ + and wild-type littermates on the 5-stimulus rCPT when treated with acute systemic modafinil and amphetamine). Data are presented as means ± SEM. Discrimination sensitivity (*d’*) is an index of the subject’s ability to distinguish target from non-target stimuli, while response criterion (*c*) describes the subject’s propensity to respond to any stimulus**. a** Modafinil: d’ and c. Modafinil caused a dose-linear decrease in d’ in the Df(h22q11)/+ model. There was no effect of modafinil on response criterion c. **b** Modafinil: hit rate and false alarm rate. Modafinil had no significant effects on hit rate or false alarm rate. **c** Amphetamine: d’ and c. Amphetamine caused a dose-linear increase in d’ in the Df(h22q11)/+ model. 1.0 mg/kg amphetamine tended to increase c in control animals only. **d** Amphetamine: hit rate and false alarm rate. Amphetamine reduced hit rate in control animals only at the 1.0 mg/kg dose. Amphetamine caused a genotype-independent reduction in hit rate at the 1.0 mg/kg dose. Pink shading denotes significant dose-linear effect that were selective to the Df(h22q11)/+ model (Ψ = *p* < 0.05). Asterisk denotes significant main effect of genotype (* = *p* < 0.05). Gray shading and hash denote significant genotype-independent dose differences (# = *p* < 0.05). Grey shading and lambda denote significant dose differences in Df(h22q11)/+ model (λ = *p* < 0.05). ‘V’ denotes drug vehicle condition (modafinil: saline + 0.5% arabic gum, amphetamine: saline)

The effect of modafinil in the rCPT is presented in Fig. 4a, b and Table 1. In Df(h22q11)/+ mice, modafinil decreased discrimination sensitivity (d’) and incorrect response latency. For d’ (Fig. 4a), there was a significant linear effect of dose (F_1,14_ = 4.947, *p* = 0.043), with modafinil dose-dependently decreasing d’. The highest 40 mg/kg dose did not significantly reduce d’ relative to vehicle (*p* = 0.075) but significantly reduced d’ relative to the 0.4 mg/kg dose (*p* = 0.021). On incorrect response latency (Table 1), there was a significant main effect of dose (F_3,42_ = 3.780, *p* = 0.017) and a significant linear effect of dose (F_1,14_ = 7.184, *p* = 0.018), with modafinil dose-dependently reducing incorrect response latency. The 40 mg/kg dose decreased incorrect response latency relative to vehicle (*p* < 0.0001) and the 4.0 mg/kg dose (*p* < 0.019).

**Table 1:**
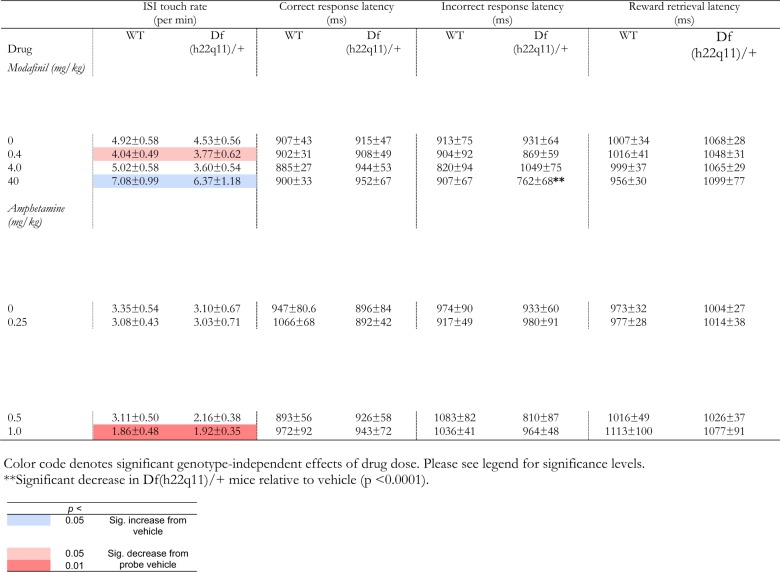
Mean response latencies and ISI touch rate of Df(h22q11) + mice and wild-types littermate controls when treated with acute systemic modafinil and acute systemic amphetamine in the rCPT

On false alarm rate (Fig. 4b), there was a significant U-shaped dose-response (F_1,14_ = 5.508, *p* = 0.034). However, post-hoc analyses comparing each dose were not significant (*p*’s ≥ 0.075). On ISI touch rate (Table 1), there was a U-shaped dose-response (F_3,42_ = 5.712, *p* = 0.031). The 40 mg/kg dose increased ISI touch rate relative to vehicle (*p* = 0.050) and the 0.04 mg/kg dose (*p* = 0.005).

#### Modafinil in both wild-type and Df(h22q11)/ + mice

When the data were speculatively analyzed across both genotypes, we observed a significant impairment in discrimination sensitivity (d’) in Df(h22q11)/+ relative to wild-type mice (Fig. 4a; F_1,26_ = 6.781, *p* = 0.015). There were no effects of modafinil on d’ (Fig. 4a). On incorrect response latency, there was a genotype × dose interaction (Table 1; genotype × dose: F_3,78_ = 3.263, *p* = 0.026). In Df(h22q11)/+ mice, the highest 40 mg/kg dose reduced incorrect response latency relative to vehicle (*p* < 0.0001). This reduction was not present in wild-type animals (*p* = 0.920).

Modafinil exerted U-shaped dose-response effects on false alarm rate across both genotypes (Fig. 4b; dose: F_3,78_ = 2.964, *p* = 0.037, quadratic effect: F_1,26_ = 5.335, *p* = 0.029). The 40 mg/kg dose significantly reduced false alarm rate relative to the 4 mg/kg dose (*p* = 0.028). Modafinil also produced U-shaped dose-responses in both genotypes on ISI touch rate (Table 1; dose: F_3,78_ = 8.128, *p* < 0.0001, quadratic effect: F_1,26_ = 6.708, *p* = 0.016). Relative to vehicle, the 0.4 mg/kg dose reduced ISI touch rate (*p* = 0.034) while the 40 mg/kg dose increased ISI touch rate (*p* = 0.015).

#### Amphetamine in the 22q11.2 model

In Df(h22q11)/+ mice, amphetamine increased discrimination sensitivity (d’). On d’ (Fig. 4c), there was a significant linear effect of dose (F_1,14_ = 6.683, *p* = 0.022) with amphetamine dose-dependently improving d’. Relative to vehicle, 1.0 mg/kg amphetamine increased d’ (*p* = 0.018).

#### Amphetamine in both wild-type and Df(h22q11)/ + mice

When the data were analyzed across both genotypes, the performance-enhancing effect of amphetamine on discrimination sensitivity (d’) was not significant (Fig. 4c; see Supplementary Table S5 for statistical analyses). However, a significant dose-linear response-reducing effects of amphetamine on ISI touch rate was observed across both genotypes (Table 1; dose: F_3,78_ = 3.793, *p* = 0.014, linear effect: F_1,26_ = 8.551, *p* = 0.007). Relative to vehicle, 1 mg/kg of amphetamine decreased ISI touch rate (*p* = 0.006).

There was also a genotype × dose interaction on hit rate (Fig. 4d; F_3,78_ = 3.037, *p* = 0.034). In wild-type animals, the highest 1.0 mg/kg dose caused a reduction in hit rate relative to the 0.5 mg/kg dose (*p* = 0.046). This reduction was not present in Df(h22q11)/+ mice (*p* = 0.848). There was a dose-linear effect of amphetamine on false alarm rate (Fig. 4d; F_1,26_ = 4.629, *p* = 0.041). Relative to vehicle, 1.0 mg/kg amphetamine decreased false alarm rates across both genotypes (*p* = 0.025). There was also a main effect of dose on response criterion (c) (Fig. 4c; F_3,78_ = 2.779, *p* = 0.047). However post-hoc analyses comparing each dose were not significant (*p*’s ≥ 0.058).

### Progressive ratio

**Fig. 5:**
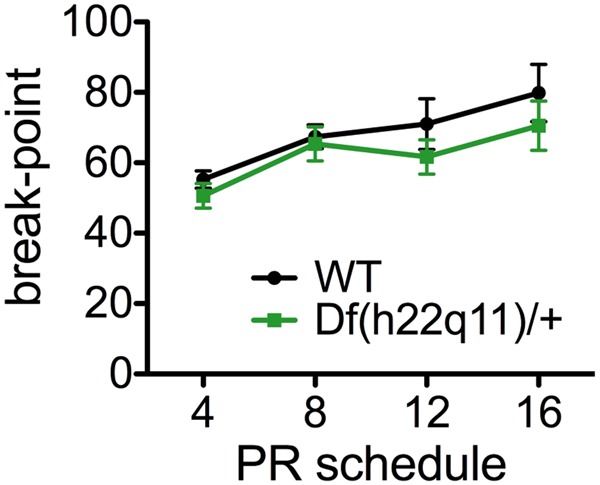
Performance of Df(h22q11)/+ and wild-types littermates on progressive ratio schedules. Data are presented as means ± SEM. There was no effect of genotype on break-point

PR performance for Df(h22q11)/+ mice and wild-type littermates is presented in Fig. 5. There was no effect of genotype on break-point (genotype: F_1,29_ = 0.882, *p* = 0.355, genotype × PR schedule: F_3,87_ = 0.603, *p* = 0.615) or any other performance measurement (*p* ≥ 0.096; data not shown).

## Discussion

The present study revealed that the 22q11.2DS mouse model (Df(h22q11)/+) exhibits neuropsychiatric disease-relevant impairments in focused visual attention. Similar rCPT impairments were observed across two cohorts that differed in age and experimental training history. These impairments in the 22q11.2DS model occurred in the absence of motivational, motoric, visual or other cognitive changes^23^, indicative of highly selective deficits in visual attentional control. Parallel with these behavioral abnormalities, the 22q11.2DS mouse model showed reduced PFC-hippocampal oscillatory synchrony in gamma, delta, and theta bands, without altered basal oscillatory activity within each region. The model deficits in discrimination sensitivity (d’) were dose-dependently improved by acute, low-dose amphetamine and, contrary to our predictions, dose-dependently impaired by acute modafinil treatment. Taken together, these data indicate a robust, selective, translationally-relevant attentional impairment in a 22q11.2DS mouse model that closely mirrors a key cognitive endophenotypic marker of 22q11.2DS and related psychiatric disorders.

### Attentional dysfunction in the Df(h22q11)/+ mouse

Attentional deficits are central to 22q11.2DS symptomatology^49^. Individuals with 22q11.2DS show CPT impairments^7,10,12,50^ and 30–40% of 22q11.2 deletion carriers are diagnosed with schizophrenia^1^ or ADHD^3^, disorders where CPT impairments represent core endophenotypes^51,52^. Individuals with 22q11.2DS, as well as with schizophrenia and ADHD, typically show decreased discrimination sensitivity (d’)^7,9^.

Deficits were observed in two separate cohorts of Df(h22q11)/+ mice that varied in both age (5 vs. 16 months at the start of testing) and previous cognitive testing experience. Although the impairments in the older cohort were somewhat less pronounced—found only under task conditions which taxed attentional load (i.e., reduced stimulus duration or increased inter-stimulus interval)—it is notable that both cohorts showed selective impairments in target hit rate. The wide age-range and relative robustness of the Df(h22q11)/+ deficits further reinforce the translational relevance of the model on the rCPT paradigm. Longitudinal studies have shown that CPT attentional impairments can persist in 22q11.2DS^7^, schizophrenia^53^, and ADHD^54^ and might represent a key endophenotypic marker of these disorders^29,55,56^.

The Df(h22q11)/+ mice appear to have cognitive deficits on the rCPT that are specific to attentional processes. We also demonstrate that Df(h22q11)/+ mice have intact motivation in a touchscreen progressive ratio task. No persistent impairment was previously observed in the model using a large cognitive testing battery^23^, and studies of cognition in alternative 22q11.2DS mouse models have generally yielded mixed results (see Table 1 in ref. ^23^). This is in apparent disparity with the clinical syndrome which has been associated with widespread and often non-selective cognitive impairment^57^. It is possible that the selective attentional impairment of the 22q11.2DS mouse model might require interaction with certain environmental risk factors to induce a more profound phenotype^58,59^. There are currently no reports assessing the effects of environmental manipulations on cognitive function in 22q11.2DS mouse models.

The mechanisms underlying impaired attention in 22q11.2DS are unknown^49^. Imaging studies of individuals with 22q11.2.DS show abnormalities within brain networks supporting attention, including structural^60,61^ and connectivity deficits^61,62^ within and between the striatum, PFC, cingulate, and temporal cortices. Structural deficits in the dorsolateral PFC and cingulate cortices of 22q11.2 deletion carriers correlate with CPT impairments^10^. Immuno-, electrophysiological- and imaging assays revealed PFC abnormalities in other 22q11.2DS models^63–65^. Such abnormalities include PFC-hippocampal theta and gamma coherence disruptions that correlate with the slower learning in a T-maze task^20,24^.

We observed similar coherence abnormalities in the current study using a 22q11.2DS model on a different background strain and employing a different recording environment (under immobile conditions). The results validate the findings of previous reports^20,24^, and additionally demonstrate that the presence of PFC-HPC asynchrony in the 22q11.2DS model is unrelated to cognitive training and ongoing behavioral performance. PFC-hippocampal synchrony aberrations may represent disrupted longer-range information integration/coordination in schizophrenia^32^, and the presence of similar disruptions in Df(h22q11)/+ mice may support its validity for evaluating genetic causes for psychopathology.

### Pharmacological effects

Modafinil and amphetamine can improve cognitive functions in humans^66–68^ and experimental animals^42,69–72^. The drugs nevertheless have both common and distinct biochemical effects^68^. The vigilance-promoting effects of amphetamine have been attributed primarily to increased dopamine/noradrenaline activity in prefrontal systems^33^, whereas modafinil has additional actions on serotonin, hypocretin/orexin, glutamate, histamine and acetylcholine functions^68^. Our data indicate that acute treatment with these drugs exerts modest but bidirectional effects on attentional performance in Df(h22q11)/+ mice.

#### Modafinil

We observed a small but significant reduction in discrimination sensitivity (d’) following acute modafinil treatment in the Df(h22q11)/+ model. This linear dose-dependent reduction in d’ was concomitant with speeding of incorrect response latency. An impairing effect of 40 mg/kg modafinil on d’ in the Df(h22q11)/+ model is similar to the observed higher-dose effects (64 mg/kg) on the rCPT in the MAM-E17 rat model of schizophrenia^31^. It is also consistent with the higher-dose effects of modafinil (64–100 mg/kg) observed in other tests of attention and/or inhibitory control, including decreased accuracy^73^ and increased premature responding in the 5-CSRTT^73,74^ and impaired Go-accuracy in the stop-signal reaction time task^43^. Low-to-moderate doses of modafinil have nevertheless been shown to improve stop-signal reaction time (10 mg/kg^43^) and CPT d’ in healthy rats (8 mg/kg)^31^ and fronto-striatal dependent cognition, including attention, in humans (100–200mg^75–77^). Unlike amphetamine, modafinil-induced improvements have generally been ascribed to enhanced inhibitory control processes that are detected in low-performing sub-groups and/or when task conditions are implemented that further challenge the ability to withhold responses^31,77^. Amphetamine has higher potency at dopamine/noradrenaline transporters than modafinil;^78^ suggesting that modafinil’s impairing effects involve additional transmitter systems. For example, modafinil, but not low-dose amphetamine, increases PFC 5-HT levels^79^. Such 5-HT increases may produce detrimental effects on impulsive-like behavior and attention when concurrent with elevations in striatal dopaminergic tone^80,81^, as displayed by the Df(h22q11)/+ model^39^.

The lack of facilitatory effects from lower-dose modafinil in the present study might be explained by the fact that, at the current task parameters, animals had low false alarm rates (~ 0.1) and thus, floor-effects could preclude detection of cognitive-enhancing effects of modafinil. By contrast, reduced baselines for false alarms would not prevent psychostimulants such as amphetamine and methylphenidate - which also enhance hit rates^38^ - from exerting performance-enhancing effects. Task parameters that challenge inhibitory control processes (e.g., variable ISIs, SDs, CS+ probabilities, flanking distractors) may be more amenable for uncovering pro-cognitive effects from low-dose modafinil in the rCPT, as was observed in the rat using variable SDs and ISIs^31^.

#### Amphetamine

We observed a significant, dose-dependent, enhancement of discrimination sensitivity (d’) in Df(h22q11)/+ animals treated with amphetamine. This improvement is in translational agreement with data showing that acute administration of the mechanistically-similar stimulant, methylphenidate, can improve d’ in children and adolescents with 22q11.2DS on a visual CPT paradigm^37^. The data are consistent with reports of low-dose stimulants improving CPT d’ in ADHD^82^ and the performances of individuals with schizophrenia on CPT-like tasks^83,84^. It is also in line with rodent studies in which low-dose amphetamine (0.25–0.5 mg/kg) or methylphenidate (2.0 mg/kg) improves attentional accuracy on serial reaction time tasks in low-attentive animals or animals challenged with short SDs^42,72,85^.

The amphetamine-induced improvements in d’ in Df(h22q11)/+ mice are due to the combined influence of increasing target hit rate and decreasing non-target false alarm rate, with neither measure showing significant changes on their own. This enhancement in d’ was accompanied by a significant reduction in the rate of extraneous touches within the response window on the screen during the ISI (in the absence of any stimuli). Together, the effects of higher discrimination sensitivity (d’) and reduced extraneous responding indicate that amphetamine dose-dependently enhances global task performance ‘efficiency’. This may reflect a unitary enhancement of attentional control or may be the result of improvements across several distinct cognitive dimensions, including attentional processing (i.e., increases in hit rate and d’) and hyperactivity/impulsivity (i.e., decreases in false alarm and ISI touch rate)^86^. Further work investigating the cognitive mechanisms underlying the performance-enhancing effects of amphetamine is warranted.

The cognitive-enhancing effects of low-dose psychostimulant treatment in the Df(h22q11)/+ model may be produced by preferential activity within the PFC, where concerted actions at noradrenaline transporters and D_1_/α_2_ receptors cause downstream glutamatergic and GABAergic events that increase neuronal tuning to behaviorally relevant stimuli^33,87^. Higher doses, however, have qualitatively different effects from lower doses and consequent deficits in signal processing^33,87^. Thus, in contrast to the clear cognitive-enhancing effects of amphetamine on rCPT performance in Df(h22q11)/+ mice, the highest dose (1.0 mg/kg) of amphetamine exerted response-suppressant effects in wild-type animals. This effect was seen as a decrease across all response rates, including hit rate, false alarm rate and the rate of ISI responses. There was an associated increase in response criterion without changes in d’. Similar suppressant effects of amphetamine have been observed in healthy well-trained rodents in the 5-CSRTT using comparable doses (≥ 0.8 mg/kg)^69,88,89^. The distinct effects of 1 mg/kg amphetamine on rCPT performance between Df(h22q11)/+ and wild-type mice (i.e., improvement rather than impairment) might be explained by the model’s hemizygosity for catecholamine-O-methyl transferase (COMT). COMT is involved in dopamine degradation primarily in regions with low expression of dopamine transporters, including the PFC^90^. In healthy rodents, higher-dose (1–1.5 mg/kg) amphetamine has potent effects on striatal relative to PFC dopamine levels^91,92^ which can have mild stimulant effects^93,94^ and alters motivational processes^95^. Decreased COMT dosage in the 22q11.2DS model could increase the ratio of PFC:striatum dopamine transmission following amphetamine treatment, resulting in increased prefrontal, task-specific, cognitive control and in fewer striatally-mediated motoric and/or motivationally-related side effects^33^. Evidence for such an altered PFC:striatum dopamine ratio has been observed in COMT^+/-^ mice after amphetamine (2.5 mg/kg) treatment, resulting in higher PFC:striatal dopamine turnover compared to wild-type controls^96^. COMT hemizygosity might additionally reduce degradation of amphetamine-induced norepinephrine release^97,98^ which, within the demanding rCPT paradigm, might also contribute to the relative absence of a response-suppressant effect of 1.0 mg/kg amphetamine in the Df(h22q11)/+ mouse.

The contrasting effects of amphetamine and modafinil on discrimination sensitivity (d’) in the rCPT might be related to differences in the chosen doses within the employed dose ranges, and/or suggest key differences in the relevant cognition-enhancing mechanisms. It would also be valuable to investigate the effects of sub-chronic/chronic dosing of these compounds to evaluate the stability of the pharmacological effects on task performance. Regardless, it is notable that both compounds influence the d’ measure, an index widely linked to attentional performance in the human CPT paradigm, selectively in Df(h22q11)/+ mice. The two drugs have been previously shown to exert neurochemical and electrophysiological modulatory effects within and between the PFC and hippocampus^33,99^. One limitation of the present study is that we did not examine drug effects on PFC-hippocampal coherence. It would be interesting to examine specific drug effects on PFC-hippocampal coherence in the Df(h22q11)/+ model—both independent of behavioral testing, and whilst systematically varying the cognitive demand of the rCPT during various dose regiments—to establish the potential role of electrophysiological correlates in our observed differences in attentional function.

### Conclusions

We demonstrate that a 22q11.2DS mouse model has selective impairments on a translationally relevant rCPT test of attention. These impairments are dose-dependently ameliorated by acute amphetamine treatment. These data closely parallel reported CPT impairments in 22q11.2 deletion carriers that are ameliorated by the psychostimulant, methylphenidate. The observed behavioral impairments were paralleled by PFC-hippocampal coherence disruptions within delta, theta and gamma bands during non-task conditions. This is the first report of attentional impairment in a 22q11.2DS model; we have demonstrated a translational utility of the Df(h22q11)/+ mouse in a fully automated and high-throughput procedure that permits large-scale and simultaneous cognitive assessment of multiple animals. In the context of the relatively limited pathology-like phenotypes that have been detected in the Df(h22q11)/+ mouse using alternative cognitive paradigms^23^, the rCPT may be a useful translational tool with enhanced sensitivity for detecting dysfunctions in rodent models.

## Electronic supplementary material

Supplemental Material (CLEAN)

Touchscreen Rodent Continuous Performance Test (rCPT) - Sample Video

Touchscreen Progressive Ratio 4 (PR4) - Sample Video
