# Supplementary material for: Continuous performance test impairment in a 22q11.2 microdeletion mouse model: improvement by amphetamine
Source: Transl Psychiatry. 2018 Nov 14;8:247. doi: 10.1038/s41398-018-0295-3 (PMC6235862; doi:10.1038/s41398-018-0295-3)
Supplement: Supplementary file 1 — Supplemental Material (CLEAN) [file 41398_2018_295_MOESM1_ESM.pdf]

SUPPLEMENTARY MATERIAL

Continuous performance test impairment in a 22q11.2 microdeletion mouse model: improvement by amphetamine

Simon RO. Nilsson, PhD[1,2,3,4], Christopher J. Heath, PhD[6], Samir Takillah, PhD[10-14], Steve Didienne, PhD[12], Kim Fejgin, PhD[5], Vibeke Nielsen, MSc[5], Jacob Nielsen, PhD[5], Lisa M. Saksida, PhD[3,4,7,8,9], Jean Mariani, MD-PhD[13,14], Philippe Faure, PhD[11], Michael Didriksen, PhD[5], Trevor W. Robbins, PhD[3,4], Timothy J. Bussey, PhD[3,4,7,8,9 ^], Adam C. Mar, PhD[1,2,3,4* ^]

^ Joint senior authors

[1]Neuroscience Institute, New York University Medical Center, New York, NY, USA. [2]Department of Neuroscience and Physiology, New York University Medical Center, New York, NY, USA. [3]Department of Psychology, University of Cambridge, Cambridge, UK. [4]MRC and Wellcome Trust Behavioural and Clinical Neuroscience Institute, University of Cambridge, Cambridge, UK. [5]H. Lundbeck A/S, Synaptic Transmission, Neuroscience Research DK, Copenhagen, Denmark. [6]School of Life, Health and Chemical Sciences, The Open University, Walton Hall, Milton Keynes, UK. [7]Molecular Medicine Research Group, Robarts Research Institute & Department of Physiology, Western University, London, ON, Canada. [8]Pharmacology, Schulich School of Medicine & Dentistry, Western University, London, ON, Canada. [9]The Brain and Mind Institute, Western University, London, ON, Canada. [10]Fatigue and Vigilance team, Neuroscience and Operational Constraints Department, French Armed Forces Biomedical Research Institute (IRBA), Brétigny-sur-Orge, France. [11]VIFASOM team (EA 7330), Paris Descartes University, Sorbonne Paris Cité, Hôtel Dieu, Paris, France. [12]Sorbonne Universités, Université Pierre et Marie Curie (UPMC), CNRS, INSERM, U1130, Institut de Biologie Paris Seine (IBPS), UMR 8246 Neuroscience Paris Seine (NPS), Team Neurophysiology and Behavior, Paris, France. [13]Sorbonne Universités, Université Pierre et Marie Curie (UPMC), CNRS, Institut de Biologie Paris Seine (IBPS), UMR 8256 Biological adaptation and ageing (B2A), Team Brain Development, Repair and Ageing, Paris France. [14]APHP Hôpital, DHU Fast, Institut de la Longévité, Ivry-Sur-Seine, France

Corresponding author: Dr Adam C Mar, Department of Neuroscience and Physiology, Neuroscience Institute, New York University Medical Center, New York, NY 10016, USA. Email: Adam.Mar@nyumc.org, Tel +1-212-263-9295

SUPPLEMENTARY METHODS

THE RODENT CONTINUOUS PERFORMANCE TEST

An 'older' cohort of of Df(h22q11)/+ mice and wild-type littermate controls (aged 16 month at start of rCPT testing; wild-type N=16, Df(h22q11)/+ N=12) that had extensive previous experience of touchscreen (5-CSRTT, paired-associate learning, extinction learning) and T-maze cognitive testing (see{Nilsson:2016cv} for data from these and other experiments) was tested on the rCPT. The previous paradigms did not reveal any persistent cognitive impairment in the Df(h22q11)/+ model. Testing one cohort of young-adult mice, and this cohort of 'older' extensively-trained mice on the rCPT, gave us opportunities to (i) gauge the relative sensitivity of the rCPT by testing Df(h22q11)/+ mice where comprehensive and clear null effects were observed in many alternative paradigms, and (ii) assess the cross-cohort reproducibility using animals differing in cognitive testing experience and age. We tested this older cohort of Df(h22q11)/+ and wild-type littermate controls on the rCPT task using probe tests of varying difficulty that are briefly described below. The probe tests consisted of manipulations to the stimulus durations (SD), target probability, inter-stimulus interval (ISI) length, and stimulus contrast:

Varying SDs. Animals were assessed in 6 separate tests of different SDs (2.5-0.25s) implemented between sessions. The limited hold (LH) was set to 2.5s at the 2.5-1.5s SDs, and 1.5s at the 1.0-0.25s SDs. The target probability was set to 50% at the 2.5-1.5s SDs, and 30% at the 1.0-0.25s SDs. The tests were presented in order of decreasing SDs. The ISI was 5s throughout.

Varying stimulus durations when controlling for event-rate. We performed two probe tests where the LH was held constant (2.5s) and the SD was manipulated (2s vs. 0.5s) across sessions. Other task parameters remained constant (ISI: 5s; CS+ probability: 30%).

Varying target probability. Animals were tested in four probe tests of decreasing target probabilities (CS+ trial probability: 50%-10%). The probe tests were implemented between sessions and presented in order of decreasing target probability with other task parameters remaining constant (SD: 2s, LH: 2.5s, ISI: 5s).

Varying ISIs. Animals were tested in three probe tests of different ISIs (5s, 10s, and 15s) implemented across sessions. The probe tests were presented in order of increasing ISI. Other task parameters remained constant (SD: 2s, LH: 2.5s, CS+ probability: 30%).

Stimuli contrast. Animals were tested in four between-session probe tests of different stimuli contrasts (100-25%). The probe tests were presented in order of decreasing stimuli contrasts. Other task parameters remained constant (SD: 1s; ISI: 5s; CS+ probability: 30%).

SUPPLEMENTARY RESULTS

THE RODENT CONTINUOUS PERFORMANCE TEST

The performances the older cohort of Df(h22q11)/+ mice and wild-type littermates is presented in Figure S1 and Table S2. Similar to the younger cohort, this older cohort of Df(h22q11)/+ animals showed impaired rCPT performance. Impairments were observed as decreased hit rates and increased response criterion c at when animals were challenged with decreased SDs and increased ISI time.

Varying SDs. The Df(h22q11)/+ mice had decreased hit rates at tests of shorter SDs (Fig. S1b; genotype: $F_{1,26}$=0.996, p=0.335, genotype × SD: $F_{5,130}$=4.795, p<0.0001). There was no effect of genotype on hit rate at the baseline rCPT test parameter (p=0.498) but the Df(h22q11)/+ had a decrease in hit rate when the SD was set to 1s (p=0.042) and non-significant decreases in hit rates at the 0.75s-0.25s SDs (p≥0.106). There was a trend for Df(h22q11)/+ mice to have increased c (Fig. S1a; genotype: $F_{1,26}$=3.749, p= 0.064, genotype × SD: $F_{5,130}$=1.312, p=0.263). Genotype did not affect *d'* (Fig. S1a; genotype: $F_{1,26}$=0.046, p=0.832, genotype × SD: $F_{5,130}$=1.100, p=0.363), or false alarm rate (Fig S1b; genotype: $F_{1,26}$=0.142, p=0.709, genotype × SD: $F_{5,130}$=0.320, p=0.900). There was no effect of genotype on ISI touch rate or latency measures in this probe tests (p≥0.152) or any other probe test (p≥0.080)(Table S2). Reducing stimulus durations decreased hit rate ($F_{5,130}$=141.887, p<0.0001), decreased false alarm rate ($F_{5,130}$=24.259, p<0.0001), decreased d' ($F_{5,130}$=29.684, p<0.0001) and increased c ($F_{5,130}$=46.075, p<0.0001) in all animals.

Varying stimulus duration when controlling for event-rate. One Df(h22q11)/+ mutant was excluded from further testing due to bad health. Decreasing the SD (from 2s to 0.5s) while holding the LH constant (2s) produced a significant genotype × SD interactions on c (genotype: $F_{1,25}$=1.397, p=0.248, genotype × SD: $F_{1,25}$=4.324, p=0.048). Reducing the SD from 2s to 0.5s increased response criterion in wild-types (p=0.015) but more so in Df(h22q11)/+ mice (p<0.0001). Accordingly, the Df(h22q11)/+ tended to show lower hit rates at the 0.5s SD, however, there effect of genotype was not significant (genotype: $F_{1,25}$=1.312, p=0.263, genotype × SD: $F_{1,25}$=1.436, p=0.242). Df(h22q11)/+ mice had

reduced false alarm rate at the 0.5s SD (genotype: $F_{1,25}$=0.914, p=0.348, genotype × SD: $F_{1,25}$=9.879, p=0.004) and *c*). There was no effect of genotype on d' ($F_{1,25}$=0.206, p=0.654, genotype × SD: $F_{1,25}$=1.241, p=0.276).

Varying target probability. Decreasing target probabilities did not show effects of genotype on hit rate (genotype: $F_{1,25}$=3.159, p=0.088, genotype × probability: $F_{3,75}$=0.474, p=0.701), false alarm rate (genotype: $F_{1,25}$=1.260, p=0.272, genotype × probability: $F_{3,75}$=0.722, p=0.542), d' (genotype: $F_{1,25}$=0.804, p=0.379, genotype × probability: $F_{3,75}$=1.026, p=0.386) or c (genotype: $F_{1,25}$=2.244, p=0.147, genotype × probability: $F_{3,75}$=0.205, p=0.892). However, lowering the target probability reduced hit rates ($F_{3,75}$=11.461, p<0.0001), reduced false alarm rates ($F_{3,75}$=4.735,p =0.004), decreased d' ($F_{3,75}$=4.550, p=0.006) and increased c ($F_{3,75}$=10.297, p<0.0001) in all animals.

Varying inter-stimulus interval. Increasing ISIs revealed an elevated response criterion c in the Df(h22q11)/+ mouse at longer ISIs (genotype: $F_{1,25}$=1.936, p=0.177; genotype × ISI: $F_{2,50}$=3.221, p=0.048). Df(h22q11)/+ mice had higher response criteria at the 15s (p=0.008) but not at the 5s (p=0.907) or 10s (p=0.25) ISIs. Genotype did not affect hit rate (genotype: $F_{1,25}$=1.142, p=0.295, genotype × ISI: $F_{2,50}$=1.728, p=0.188) false alarm rate (genotype: $F_{1,25}$=2.049, p=0.165, genotype × ISI: $F_{2,50}$=0.562, p=0.574) or d' (genotype: $F_{1,25}$=0.072, p=0.790, genotype × ISI: $F_{2,50}$=0.652, p=0.526). Longer ISI increase in false alarm rate ($F_{2,50}$=5.767, p=0.006) in all animals.

Varying stimulus contrasts. Decreasing the stimulus contrast produced no effect of genotype on hit rate (genotype: $F_{1,25}$=1.588, p=0.219, genotype × contrast: $F_{3,75}$=0.359, p=0.783), false alarm rate (genotype: $F_{1,25}$=1.197, p=0.284, genotype × contrast: $F_{3,75}$=1.764, p=0.161), c (genotype: $F_{1,25}$=2.140, p=0.156, genotype × contrast: $F_{3,75}$=0.265, p=0.851) or d' (genotype: $F_{1,25}$=0.273, p=0.606, genotype × contrast: $F_{3,75}$=0.970, p=0.412). However, lowering stimuli contrasts increase false alarm rate ($F_{3,75}$=36.379, p<0.0001), decreased d' ($F_{3,75}$=27.211, p<0.0001), and decreased c ($F_{3,75}$=9.456, p<0.0001) and in all animals.

SUPPLEMENTARY TABLE S1

Table S1. Statistical analyses of drug-free rCPT performance in WT and Df(h22q11)/+ mice. Significant effects are shaded in pink.

	Mean±SEM					
	WT	Df(h22q11)/+	factor	F	df	***p***
Stage 1						
Hit rate	0.96 ± 0.02	0.96 ± 0.01		0.23	1,26	0.881
Response latency (ms)	3.59 ± 0.19	4.09 ± 0.27	genotype	2.107	1,26	0.159
Reward retrieval latency (ms)	1.96 ± 0.07	1.69 ± 0.11		3.980	1,26	0.057
Session completion time (s)	479 ± 30	494 ± 24		0.698	1,26	0.698
Stage 2 – target stimulus only						
Hit rate	0.96 ± 0.01	0.92 ± 0.03		1.981	1,26	0.171
ISI touch rate	1.01 ± 0.28	0.88 ± 0.12		0.193	1,26	0.664
Response latency (ms)	1.92 ± 0.41	2.31 ± 0.26	genotype	0.653	1,26	0.426
Reward retrieval latency (ms)	1.49 ± 0.07	1.37 ± 0.08		1.066	1,26	0.311
Session completion time (s)	817 ± 22	862 ± 27		1.560	1,26	0.223
Stage 3 – Single non-target						
Discrimination sensitivity (d’)	1.56±0.12	1.20±0.13	genotype	4.203	1,26	0.051
			genotype × session	0.816	4,104	0.518
Response criterion (c)	-0.24±0.04	-0.02±0.07	genotype	6.971	1,26	0.014
			genotype × session	1.107	4,104	0.357
Hit rate	0.82±0.02	0.70±0.03	genotype	9.552	1,26	0.005
			genotype × session	0.744	4,104	0.564
False alarm rate	0.32±0.02	0.30±0.03	genotype	0.288	1,26	0.596
			genotype × session	1.670	4,104	0.162
ISI touch ratio	0.17±0.03	0.19±0.01	genotype	0.611	1,26	0.442
			genotype × session	1.302	4,104	0.302
Correct response latency (ms)	1016±36	1024±25	genotype	0.035	1,26	0.853
Incorrect response latency (ms)	945±34	953±21	genotype	0.050	1,26	0.825
Reward retrieval latency (ms)	1194±43	1210±59	genotype	0.048	1,26	0.829
Baseline rCPT						
Discrimination sensitivity (d’)	2.41±0.08	2.07±0.12	genotype	5.724	1,26	0.030
			genotype × session	0.932	5,130	0.462
Response criterion (c)	0.62±0.07	0.65±0.08	genotype	0.197	1,26	0.661
			genotype × session	0.260	5,130	0.934
Hit rate	0.88±0.01	0.80±0.03	genotype	4.578	1,26	0.042
			genotype × session	0.333	5,130	0.892
False alarm rate	0.13±0.01	0.14±0.01	genotype	0.664	1,26	0.423
			genotype × session	0.519	5,130	0.761
ISI touch ratio	0.09±0.01	0.09±0.01	genotype	0.262	1,26	0.613
			genotype × session	0.548	5,130	0.740
Correct response latency (ms)	909±31	918±46	genotype	0.026	1,26	0.873
Incorrect response latency (ms)	898±29	888±31	genotype	0.053	1,26	0.820
Reward retrieval latency (ms)	998±29	1004±32	genotype	0.022	1,26	0.882

SUPPLEMENTARY TABLES S2-3

Table S2. Statistical analyses of modafinil in the Df(h22q11)/+ model. Significant effects are shaded in pink.

	Mean (±SEM)	factor	F	df	P
Discrimination sensitivity (d')					
0	2.40±0.18	Dose	2.469	3,42	0.075
0.4	2.55±0.20	Dose linear effect	4.947	1,14	0.043
4.0	2.66±0.22	Dose quadratic effect	1.704	1,14	0.213
40	2.72±0.19				
Response criterion (c)					
0	0.18±0.11	dose	0.865	3,42	0.405
0.4	0.15±0.12	dose linear effect	0.990	1,14	0.337
4.0	0.19±0.07	Dose quadratic effect	0.822	1,14	0.380
40	0.19±0.13				
Hit rate					
0	0.81±0.04	dose	1.025	3,42	0.348
0.4	0.83±0.04	Dose linear effect	2.187	1,14	0.161
4.0	0.84±0.03	Dose quadratic effect	0.028	1,14	0.870
40	0.84±0.04				
False alarm rate					
0	0.10±0.02	Dose	2.375	3,42	0.084
0.4	0.11±0.03	Dose linear effect	0.803	1,14	0.385
4.0	0.08±0.02	Dose quadratic effect	5.508	1,14	0.034
40	0.09±0.02				
ISI touch rate					
0	0.05±0.01	Dose	3.561	3,42	0.600
0.4	0.05±0.01	Dose linear effect	2.041	1,14	0.175
4.0	0.04±0.01	Dose quadratic effect	5.712	1,14	0.031
40	0.03±0.01				
Correct response latency (ms)					
0	896±84	Dose	0.568	3,42	0.639
0.4	892±42	Dose linear effect	0.957	1,14	0.344
4.0	927±59	Dose quadratic effect	0.074	1,14	0.790
40	943±72				
Incorrect response latency (ms)					
0	934±60	Dose	3.780	3,42	0.017
0.4	981±91	Dose linear effect	7.184	1,14	0.018
4.0	810±87	Dose quadratic effect	1.357	1,14	0.263
40	964±48				
Reward retrieval latency (ms)					
0	1004±28	Dose	0.294	3,42	0.830
0.4	1014±38	Dose linear effect	0.254	1,14	0.622
4.0	1026±38	Dose quadratic effect	0.417	1,14	0.529
40	1077±91				

Table S3. Statistical analyses of amphetamine in the Df(h22q11)/+ model. Significant effects are shaded in pink.

	Mean (±SEM)	factor	F	df	p
Discrimination sensitivity (d')					
0	2.40±0.18	Dose	1.903	3,42	0.143
0.4	2.55±0.20	Dose linear effect	6.683	1,14	0.022
4.0	2.66±0.22	Dose quadratic effect	0.164	1,14	0.960
40	2.72±0.19				
Response criterion (c)					
0	0.18±0.11	dose	0.061	3,42	0.980
0.4	0.15±0.12	dose linear effect	0.055	1,14	0.818
4.0	0.19±0.07	Dose quadratic effect	0.022	1,14	0.884
40	0.19±0.13				
Hit rate					
0	0.81±0.04	dose	0.465	3,42	0.708
0.4	0.83±0.04	Dose linear effect	1.864	1,14	0.194
4.0	0.84±0.03	Dose quadratic effect	0.248	1,14	0.626
40	0.84±0.04				
False alarm rate					
0	0.10±0.02	Dose	0.696	3,42	0.560
0.4	0.11±0.03	Dose linear effect	1.380	1,14	0.260
4.0	0.08±0.02	Dose quadratic effect	0.053	1,14	0.821
40	0.09±0.02				
ISI touch rate					
0	0.05±0.01	Dose	1.758	3,42	0.170
0.4	0.05±0.01	Dose linear effect	4.248	1,14	0.058
4.0	0.04±0.01	Dose quadratic effect	0.038	1,14	0.849
40	0.03±0.01				
Correct response latency (ms)					
0	896±84	Dose	0.152	3,42	0.928
0.4	892±42	Dose linear effect	0.283	1,14	0.603
4.0	927±59	Dose quadratic effect	0.024	1,14	0.880
40	943±72				
Incorrect response latency (ms)					
0	934±60	Dose	1.487	3,42	0.232
0.4	981±91	Dose linear effect	0.078	1,14	0.784
4.0	810±87	Dose quadratic effect	0.523	1,14	0.481
40	964±48				
Reward retrieval latency (ms)					
0	1004±28	Dose	0.436	3,42	0.728
0.4	1014±38	Dose linear effect	0.946	1,14	0.347
4.0	1026±38	Dose quadratic effect	0.120	1,14	0.795
40	1077±91				

SUPPLEMENTARY TABLES S4-5

Table S4. Statistical analyses of modafinil on rCPT performance in WT and Df(h22q11)/+ mice. Significant effects are shaded in pink.

	Mean ±SEM WT	Df (h22q11)/+	Factor	F	df	p
Discrimination sensitivity (d')						
0	2.63±0.10	2.28±0.13	Genotype	6.781	1,26	0.015
0.4	2.58±0.11	2.33±0.15	Dose	1.825	3,78	0.149
4.0	2.71±0.09	2.21±0.14	Genotype × dose	1.052	3,78	0.374
40	2.55±0.14	2.01±0.18				
Response criterion (c)						
0	-0.03±0.07	0.02±0.08	Genotype	2.055	1,26	0.164
0.4	0.01±0.06	0.11±0.09	Dose	0.613	3,78	0.609
4.0	-0.03±0.06	0.23±0.09	Genotype × dose	0.619	3,78	0.605
40	-0.01±0.09	0.13±0.16				
Hit rate						
0	0.90±0.01	0.85±0.03	Genotype	6.155	1,26	0.020
0.4	0.89±0.01	0.83±0.04	Dose	0.876	3,78	0.457
4.0	0.91±0.01	0.79±0.03	Genotype × dose	0.829	3,78	0.482
40	0.90±0.02	0.78±0.06				
False alarm rate						
0	0.11±0.02	0.14±0.02	Genotype	0.708	1,26	0.408
0.4	0.11±0.02	0.12±0.02	Dose	2.964	3,78	0.037
4.0	0.10±0.01	0.11±0.02	Genotype × dose	0.461	3,78	0.710
40	0.13±0.02	0.17±0.04	Dose quadratic effect	5.335	1,26	0.029
ISI touch rate						
0	0.08±0.01	0.08±0.01	Genotype	0.812	1,26	0.376
0.4	0.07±0.01	0.06±0.01	Dose	8.128	3,78	>0.0001
4.0	0.08±0.01	0.06±0.01	Genotype × dose	0.341	3,78	0.769
40	0.12±0.02	0.11±0.02				
Correct response latency (ms)						
0	907±43	916±47	Genotype	0.287	1,26	0.597
0.4	902±31	908±49	Dose	0.237	3,78	0.871
4.0	885±27	944±53	Genotype × dose	0.583	3,78	0.628
40	900±33	952±67				
Incorrect response latency (ms)						
0	914±75	931±64	Genotype	0.052	1,26	0.821
0.4	904±92	869±59	Dose	1.075	3,78	0.365
4.0	820±94	1050±75	Genotype × dose	3.263	3,78	0.026
40	907±67	762±68				
Reward retrieval latency (ms)						
0	1007±34	1068±28	Genotype	2.722	1,26	0.111
0.4	1017±41	1048±31	Dose	0.034	3,78	0.992
4.0	999±37	1065±30	Genotype × dose	1.153	3,78	0.333
40	956±30	1099±77				

Table S5. Statistical analyses of amphetamine on rCPT performance in WT and Df(h22q11)/+ mice. Significant effects are shaded in pink.

	Mean ±SEM WT	Df (h22q11)/+	Factor	F	df	p
Discrimination sensitivity (d')						
0	2.78±0.13	2.40±0.18	Genotype	1.693	1,26	0.205
0.4	2.92±0.12	2.55±0.20	Dose	0.977	3,78	0.408
4.0	2.88±0.16	2.66±0.22	Genotype × dose	0.563	3,78	0.641
40	2.83±0.21	2.72±0.19				
Response criterion (c)						
0	0.21±0.09	0.18±0.11	Genotype	0.560	1,26	0.461
0.4	0.16±0.08	0.15±0.12	Dose	2.779	3,78	0.047
4.0	0.16±0.07	0.19±0.07	Genotype × dose	2.304	3,78	0.083
40	0.56±0.22	0.19±0.13				
Hit rate						
0	0.89±0.01	0.81±0.04	Genotype	0.724	1,26	0.403
0.4	0.90±0.01	0.83±0.04	Dose	2.503	3,78	0.063
4.0	0.90±0.02	0.84±0.03	Genotype × dose	3.037	3,78	0.034
40	0.76±0.07	0.84±0.04				
False alarm rate						
0	0.07±0.01	0.10±0.02	Genotype	2.676	1,26	0.114
0.4	0.07±0.01	0.11±0.03	Dose	1.444	3,78	0.236
4.0	0.07±0.01	0.08±0.02	Genotype × dose	0.917	3,78	0.437
40	0.04±0.01	0.09±0.02	Dose linear effect	4.629	1,26	0.041
ISI touch rate						
0	0.06±0.01	0.05±0.01	Genotype	0.309	1,26	0.583
0.4	0.05±0.01	0.05±0.01	Dose	3.793	3,78	0.014
4.0	0.05±0.01	0.04±0.01	Genotype × dose	0.549	3,78	0.650
40	0.03±0.01	0.03±0.01	Dose linear effect	8.551	1,26	0.007
Correct response latency (ms)						
0	947±81	896±84	Genotype	1.006	1,26	0.325
0.4	1066±68	892±42	Dose	0.435	3,78	0.728
4.0	894±56	927±59	Genotype × dose	0.812	3,78	0.491
40	972±92	943±72				
Incorrect response latency (ms)						
0	974±90	934±60	Genotype	1.490	1,26	0.233
0.4	917±49	981±91	Dose	0.316	3,78	0.813
4.0	1083±82	810±87	Genotype × dose	2.451	3,78	0.070
40	1036±41	964±48				
Reward retrieval latency (ms)						
0	973±33	1004±28	Genotype	0.044	1,26	0.835
0.4	977±28	1014±38	Dose	1.844	3,78	0.146
4.0	1016±49	1026±38	Genotype × dose	0.213	3,78	0.887
40	1113±100	1077±91				

SUPPLEMENTARY TABLE S6

Table S6. Performance of Df(h22q11)/+ and wild-type littermates (older cohort) when challenged with probe test of varying difficulty.

	d’		c		hit rate		false alarm rate		ISI touch rate (per min)		Correct latency (ms)		Incorrect latency (ms)		Retrieval latency (ms)	
Parameter	WT	Df (h22q11)/+	WT	Df (h22q11)/+	WT	Df (h22q11)/+	WT	Df (h22q11)/+	WT	Df (h22q11)/+	WT	Df (h22q11)/+	WT	Df (h22q11)/+	WT	Df (h22q11)/-
SD (target %)																
2.5s (50)	1.85±0.17	1.94±0.20	0.02±0.10	0.02±0.08	0.80±0.04	0.83±0.03	0.21±0.04	0.21±0.03	4.40±0.84	5.46±0.91	986±43	921±41	970±64	1049±93	1057±38	1016±46
1.5s (50)	1.89±0.12	2.02±0.20	0.29±0.10	0.22±0.13	0.73±0.04	0.77±0.05	0.13±0.03	0.15±0.05	5.19±1.74	6.50±.2.66	859±41	903±49	848±46	752±53	1104±50	1061±43
1.0 (30)	1.95±0.10	1.76±0.12	0.60±0.07	0.86±0.11	0.64±0.04	0.51±0.05	0.07±0.01	0.06±0.02	3.43±0.33	3.08±0.41	1007±41	1024±33	944±41	939±40	1084±44	1080±40
0.75 (30)	1.70±0.12	1.65±0.14	0.75±0.08	0.91±0.10	0.54±0.04	0.47±0.05	0.07±0.01	0.05±0.01	3.59±0.57	3.06±0.47	636±21	633±24	654±22	603±26	1196±86	1178±64
0.5 (30)	1.43±0.11	1.44±0.13	0.83±0.07	1.13±0.10	0.46±0.04	0.36±0.05	0.07±0.01	0.04±0.01	3.71±0.36	3.16±0.46	578±26	626±57	656±18	582±50	1089±42	1046±53
0.25 (30)	1.12±0.10	0.92±0.18	0.97±0.06	1.22±0.14	0.35±0.03	0.28±0.05	0.07±0.01	0.06±0.01	4.60±0.54	3.89±0.68	568±34	560±23	645±22	623±33	1117±36	1108±39
SD (when controlling event rate)																
2s SD	2.41±0.12	2.25±0.12	0.30±0.10	0.34±0.08	0.80±0.03	0.77±0.03	0.09±0.03	0.08±0.01	3.62±0.72	2.77±0.43	1063±54	1051±45	1291±126	1130±37	1175±53	1130±37
0.5s SD	1.44±0.14	1.49±0.16	0.47±0.08	0.69±0.06	0.60±0.04	0.52±0.05	0.13±0.02	0.08±0.01	4.89±0.78	4.02±0.51	991±43	1008±46	1205±54	1096±43	1205±54	1096±43
Target %																
50%	2.44±0.10	2.40±0.13	0.34±0.09	0.53±0.10	0.79±0.03	0.73±0.04	0.08±0.02	0.05±0.01	2.89±0.36	2.95±0.64	694±26	659±33	599±37	576±35	1066±51	1045±31
30%	2.34±0.08	2.30±0.12	0.27±0.08	0.39±0.07	0.81±0.02	0.76±0.03	0.09±0.02	0.07±0.01	4.02±0.44	3.93±0.60	687±22	699±21	693±20	683±34	1118±57	1059±38
20%	2.32±0.10	2.06±0.15	0.43±0.08	0.60±0.08	0.75±0.03	0.65±0.05	0.07±0.01	0.06±0.01	4.24±0.62	3.44±0.58	689±26	692±32	695±51	651±29	1113±41	1063±31
10%	2.26±0.09	2.13±0.13	0.58±0.07	0.69±0.08	0.70±0.03	0.63±0.05	0.05±0.01	0.05±0.01	2.07±0.33	2.57±0.48	1079±36	1135±41	1085±75	1196±68	1129±53	1069±40
ISI																
5s	2.50±0.18	2.44±0.13	0.46±0.10	0.47±0.08	0.76±0.04	0.76±0.04	0.06±0.01	0.05±0.01	1.92±0.33	1.91±0.34	1023±46	1044±43	973±100	1223±110	1222±78	1078±30
10s	2.31±0.15	2.40±0.21	0.29±0.08	0.39±0.06	0.79±0.03	0.76±0.04	0.09±0.02	0.06±0.01	2.47±0.39	2.14±0.42	984±38	991±56	1129±89	981±64	1215±67	1103±54
15s	2.40±0.12	2.23±0.17	0.15±0.07	0.46±0.08	0.84±0.02	0.73±0.04	0.10±0.02	0.07±0.01	2.75±0.48	2.40±0.39	943±34	1000±39	1098±66	1126±93	1116±61	1103±42
Contrast																
100%	2.18±0.19	1.98±0.20	0.79±0.12	0.96±0.10	0.58±0.05	0.51±0.05	0.06±0.01	0.05±0.01	2.91±0.50	3.16±0.65	695±32	670±24	677±19	594±22	1136±55	1095±43
75%	1.82±0.16	1.67±0.21	0.69±0.14	0.90±0.09	0.55±0.05	0.47±0.04	0.09±0.02	0.07±0.01	3.32±0.47	3.21±0.92	761±41	748±28	848±58	878±50	1071±48	1004±23
50%	1.82±0.15	1.61±0.23	0.50±0.10	0.77±0.11	0.62±0.04	0.50±0.06	0.10±0.01	0.09±0.01	3.65±0.37	3.46±0.54	753±34	787±26	848±21	817±42	1355±233	1071±44
25%	1.27±0.15	1.32±0.19	0.47±0.11	0.67±0.10	0.54±0.05	0.49±0.05	0.15±0.02	0.11±0.02	4.17±0.50	4.02±0.57	785±35	793±32	783±25	819±37	1100±43	1075±40

Significant effects of task parameter manipulation and task parameter × genotype interactions denoted by colour codes. For significance level, please see legend.

	$p <$	
(light blue)	0.05	Sig. increase from probe baseline
(medium blue)	0.01	
(blue)	0.001	
(light red)	0.05	Sig. decrease from probe baseline
(medium red)	0.01	
(red)	0.001	
(green)	0.05	Sig. genotype × parameter interaction. Sig. different from wild-type.

SUPPLEMENTARY FIGURE S1

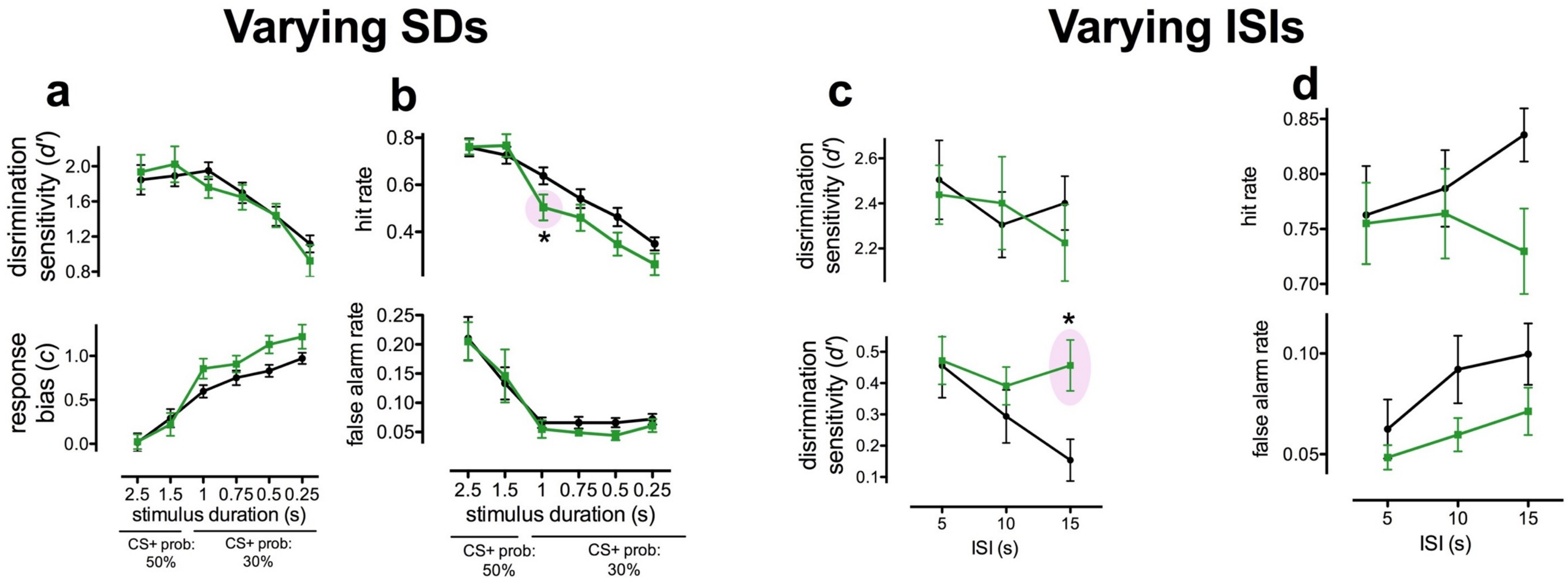

Figure S1. Performance of Df(h22q11)/+ and wild-type littermates (older, extensively trained, cohort) when challenged with shorter SDs (a-b) and longer ISIs (c-d). Discrimination sensitivity (*d'*) is an index of the subject's ability to distinguish target from non-target stimuli, while response criterion (*c*) describes the subject's propensity to respond to any stimulus **(a) Varying SDs: d' and c.** No significant effect of genotype or genotype × SD interaction on either d' or c. **(b) Varying SDs: hit rate and false alarm rate.** Df(h22q11)/+ mice had reduced hit rate at shorter stimulus durations. There was no effect of genotype on false alarm rate. **(c)Varying ISIs: d' and c.** Df(h22q11)/+ mice had elevated response criterion c at longer ISIs relative to littermate controls. There was no effect of genotype on d'. **(d) Varying ISIs: hit rate and false alarm rate.** Df(h22q11)/+ mice tended to show reduced hit rate at the longer ISI. There was no significant effect of genotype on hit rate or false alarm rate.

SUPPLEMENTARY FIGURE S2

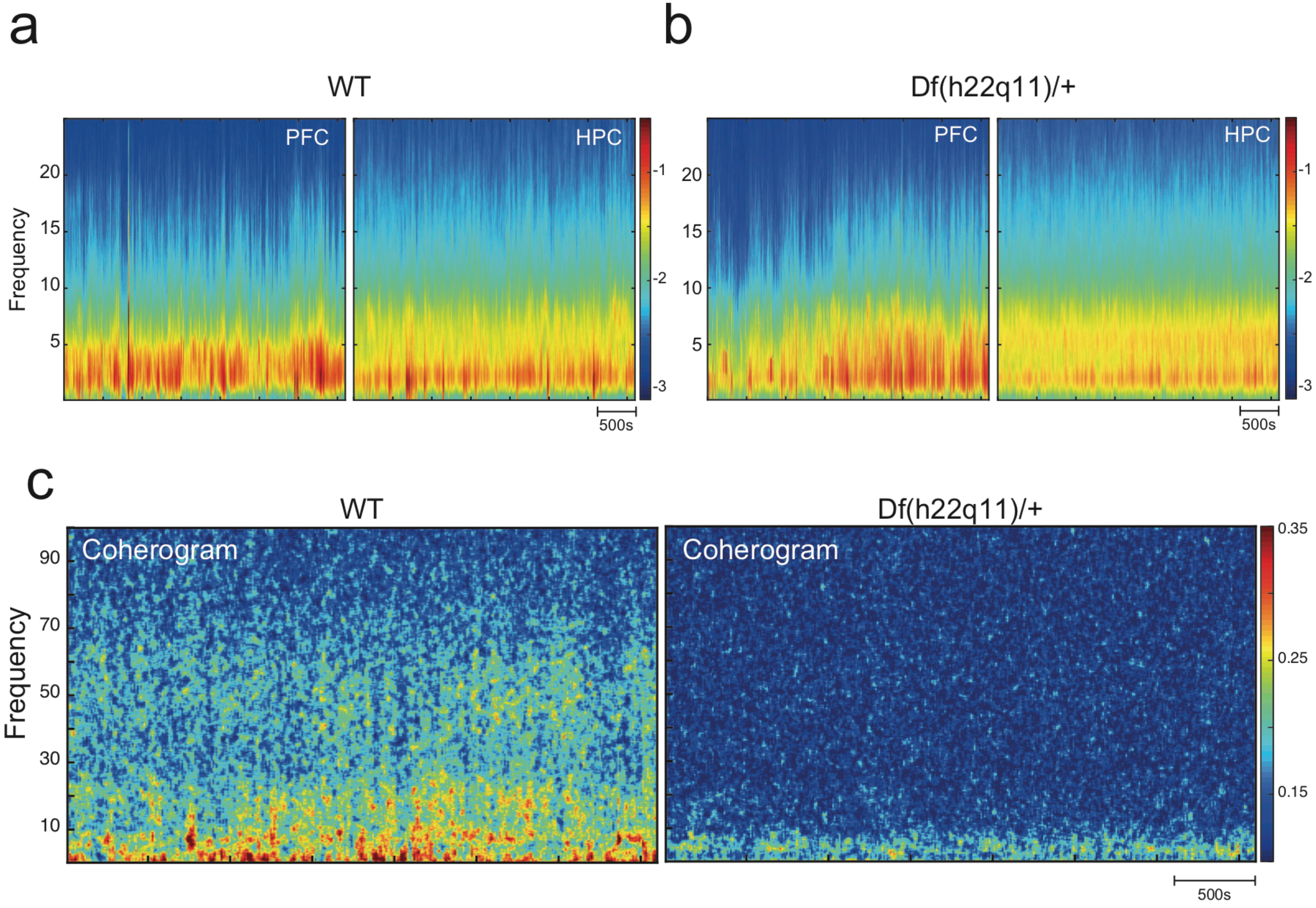

Figure S2. Spectrogram of PFC and dorsal HPC during steady state conditions in (a) WT and (b) Df(22q11.2)/+ animals. (c) Coherograms (PFC-dHPC coherence over the time) in WT and (b) Df(22q11.2)/+ animals.

SUPPLEMENTARY FIGURE S3

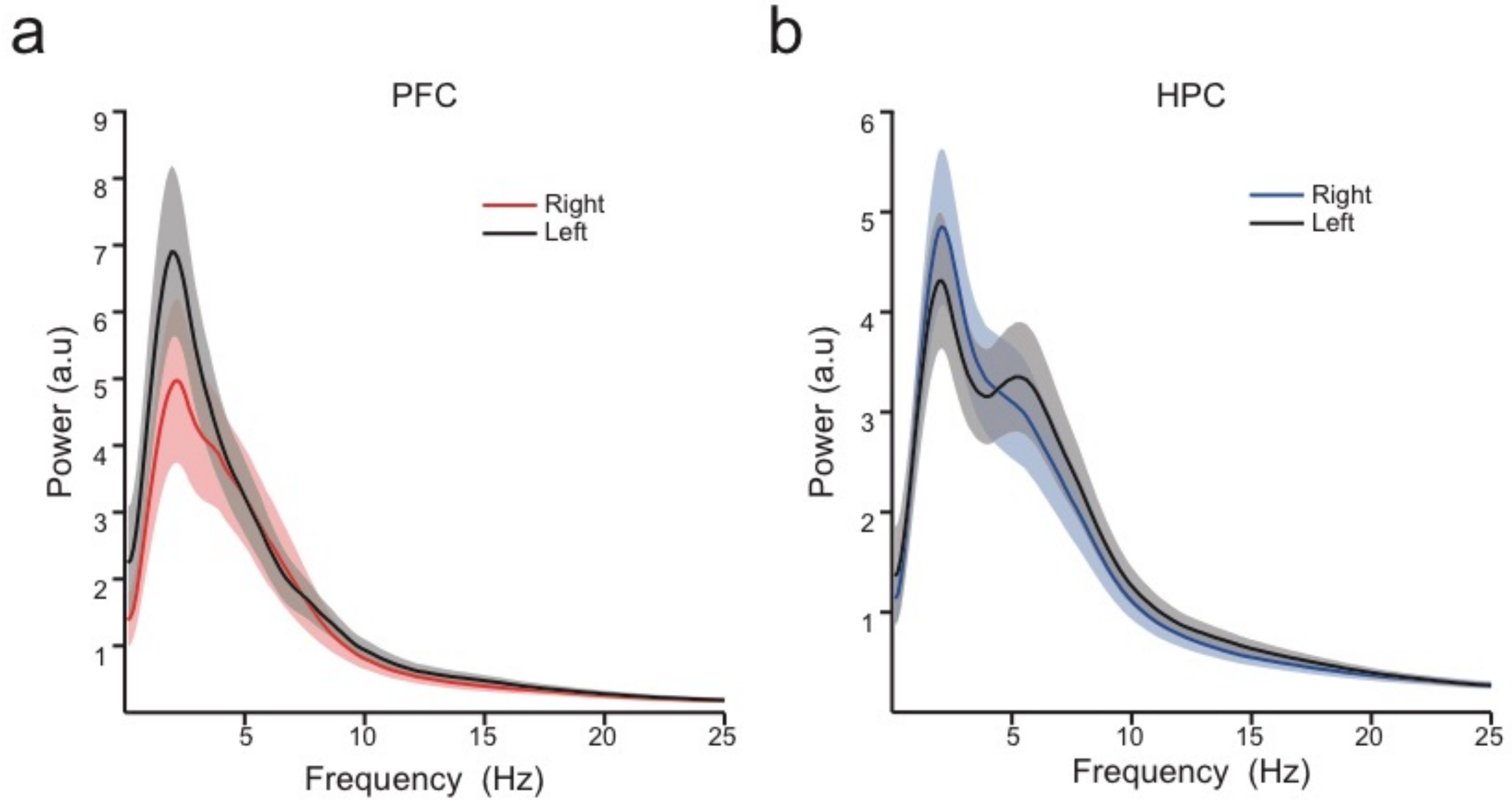

Figure S3. WT: Averaged power spectrum for each hemisphere (left, right) (a) in the PFC and (b) in the HPC.

SUPPLEMENTARY FIGURE S4

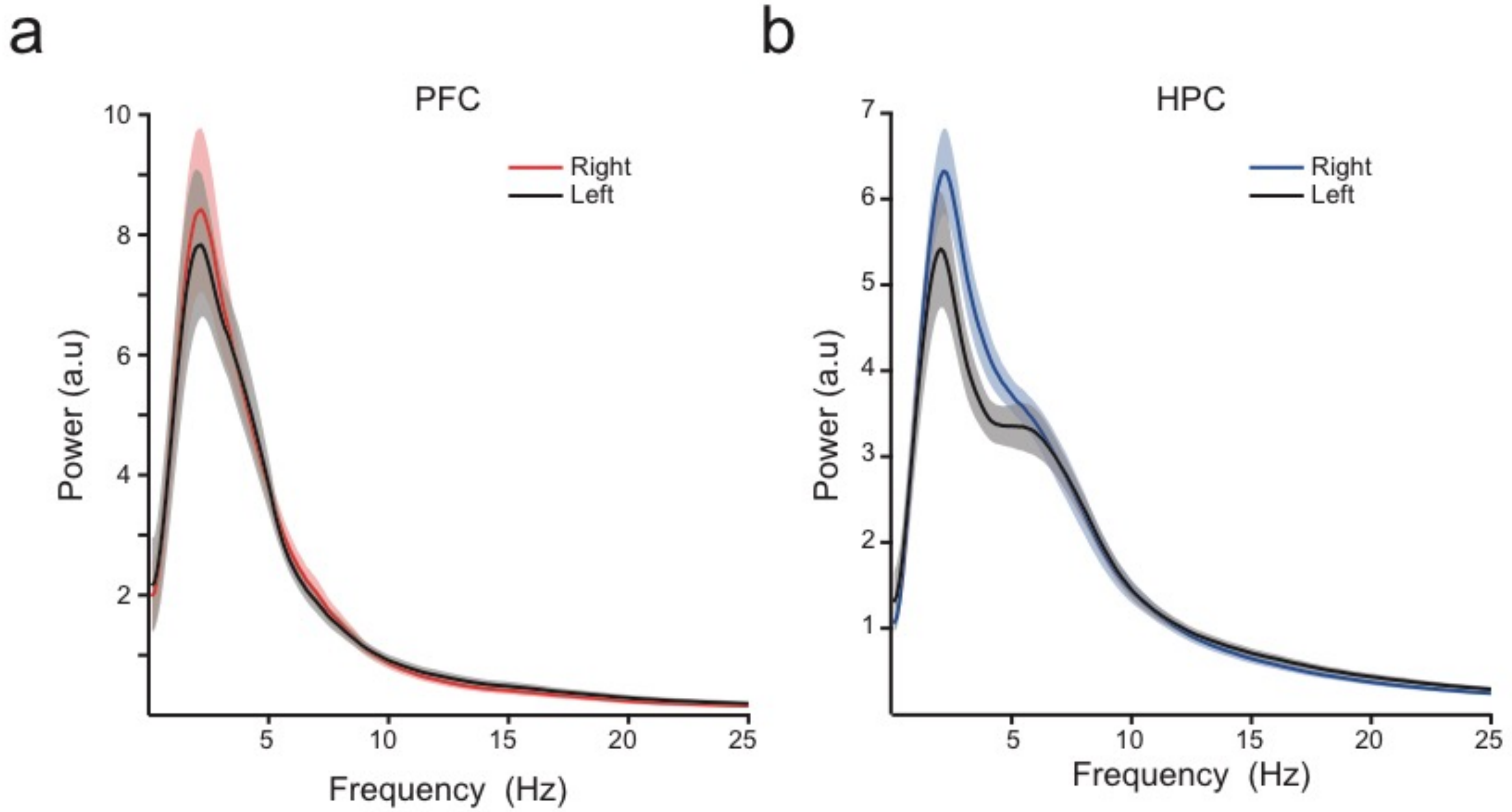

Figure S4. Df(22q11.2)/+: Averaged power spectrum for each hemisphere (left, right) (a) in the PFC and (b)in the HPC.
